# Caspase-Based Fusion Protein Technology: Substrate Cleavability Described by Computational Modeling and Simulation

**DOI:** 10.1021/acs.jcim.4c00316

**Published:** 2024-07-01

**Authors:** Jakob Liu, Andreas Fischer, Monika Cserjan-Puschmann, Nico Lingg, Chris Oostenbrink

**Affiliations:** † Austrian Centre of Industrial Biotechnology, Muthgasse 18, 1190 Vienna, Austria; ‡ Institute of Molecular Modeling and Simulation, University of Natural Resources and Life Sciences, Vienna (BOKU), Muthgasse 18, 1190 Vienna, Austria; § Department of Biotechnology, Institute of Bioprocess Science and Engineering, University of Natural Resources and Life Sciences, Vienna (BOKU), Muthgasse 18, 1190 Vienna, Austria; ∥ Christian Doppler Laboratory for Molecular Informatics in the Biosciences, University of Natural Resources and Life Sciences, Vienna, Muthgasse 18, 1190 Vienna, Austria

## Abstract

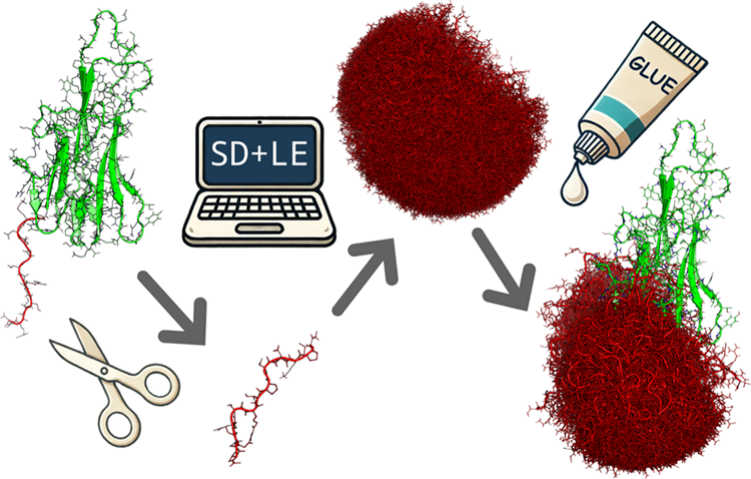

The Caspase-based fusion protein technology (CASPON) allows for universal cleavage of fusion tags from proteins of interest to reconstitute the native N-terminus. While the CASPON enzyme has been optimized to be promiscuous against a diversity of N-terminal peptides, the cleavage efficacy for larger proteins can be surprisingly low. We develop an efficient means to rationalize and predict the cleavage efficiency based on a structural representation of the intrinsically disordered N-terminal peptides and their putative interactions with the CASPON enzyme. The number of favorably interacting N-terminal conformations shows a very good agreement with the experimentally observed cleavage efficiency, in agreement with a conformational selection model. The method relies on computationally cheap molecular dynamics simulations to efficiently generate a diverse collection of N-terminal conformations, followed by a simple fitting procedure into the CASPON enzyme. It can be readily used to assess the CASPON cleavability a priori.

## Introduction

In recent decades, recombinant proteins have become an essential player in the pharmaceutical industry, greatly improving treatment outcomes for patients.^1^ Their production typically takes place in bacterial, fungal, or animal cell factories, with one of the most resource-intensive steps being the separation of the desired protein of interest (POI) from the crude media solution.^2−4^ This downstream process generally uses several filtration, centrifugation, and chromatographic purification steps. The complexity and number of required unit operations, which may need to be tailored to each specific POI, leads to increased development time and manufacturing costs.^2^

Affinity tags offer a way to circumvent this problem.^5−7^ A notable example would be a polyhistidine tag (His-tag), used in tandem with immobilized metal affinity chromatography (IMAC).^7^ The POI can be produced including such a tag at either the N- or C-terminus. The purification of such fusion proteins is substantially facilitated by leveraging the robustness and versatility of the IMAC method, which allows for specific and efficient capture of His-tagged proteins, resulting in high purity and recovery yield due to the specific and reversible interaction between the histidines of the His-tag and immobilized metal ions in the stationary phase of the chromatography column.^8^ Remaining impurities due to natural affinity for the column are reasonably well understood.^9^ Furthermore, the tags can easily be combined with other tags that enhance the solubility and expression of the POI.^10−14^

When producing the POI for pharmaceutical applications, it is usually necessary that the tag is cleaved after successful purification to restore the native N-terminus and to prevent any potential interference with the biological function of the POI or undesired immune responses against the tag.^10,15−17^ One way to achieve this is the use of a protease that specifically cleaves at the designated cleavage site and separates the tag and the POI without any overhanging residues.^13^

To successfully adopt such a system for a wide range of proteins, the protease must exhibit high specificity toward the tag, while being promiscuous to the N-terminus of the POI. The latter is particularly difficult to achieve due to the distinctive structure, function, and behavior of each protein, usually making proteases specific toward both sides of the cleavage site.^10^

**Figure 1:**
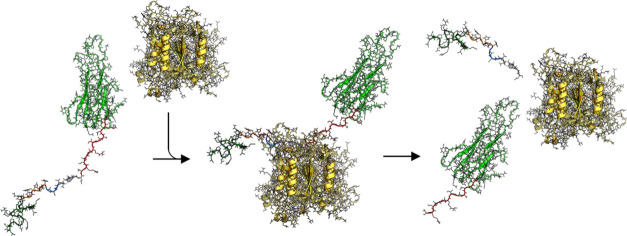
Fusion protein technologies aid in the expression and purification of recombinant proteins (green) but require specific proteases for tag removal. The CASPON enzyme (yellow), which is a modified version of circularly permutated caspase-2 (cpCasp2), with its high specificity on the N-terminal side and high promiscuity on the C-terminal side of the cleavage site, presents itself as a versatile solution for tag removal across diverse protein sequences. By efficiently binding to the designated recognition site and cleaving the tag, the CASPON enzyme restores the untagged POI by restoring its native N-terminus (red).

The circularly permuted version of human caspase-2 (cpCasp2)^18^ represents such a universally applicable protease; see Figure 1. This altered version of human caspase-2 efficiently yields untagged protein. The circular permutation allows for an immediate activation of the enzyme, which distinguishes it from human caspase-2. The wild-type enzyme originally is a zymogen that requires cleavage on itself to transition to its active dimeric state. The cpCasp2 is easily produced in *Escherichia coli*, a beneficial characteristic of a protease that is intended for large-scale use. Notably, its expression in *E. coli* has improved in comparison to that of wild-type caspase-2. This enzyme excels in activity and manufacturability, outperforming similar proteases while maintaining high specificity and facilitating the generation of the native N-terminus of POIs without off-target cleavage or requiring a large tag. However, the cleavage efficiency of it is strongly dependent on the C-terminal side of the cleavage site, termed the P1′ site. This was tested by cleaving a tag from small peptides. Each small peptide had the same sequence, varying only in the P1′ position to test all 20 proteogenic amino acids. Its catalytic rate declines when cleavage occurs prior to branched (valine, leucine, isoleucine) and acidic (aspartate, glutamate) amino acids and is even slower before proline.^13^

The same team later introduced an upgraded version of cpCasp2, integrating four point mutations. Two of these, E105V and G171D, are in flexible loops close to the active site and augment the enzymatic activity. In particular, the cleavage activity for challenging amino acids at the P1′ position was considerably increased, with a 50-fold improvement in the case of proline. The other two mutations, V225G and D282E, at more solvent-exposed locations did not influence enzymatic activity but further optimized the soluble expression of the improved cpCasp2 variant.^13^

**Figure 2:**
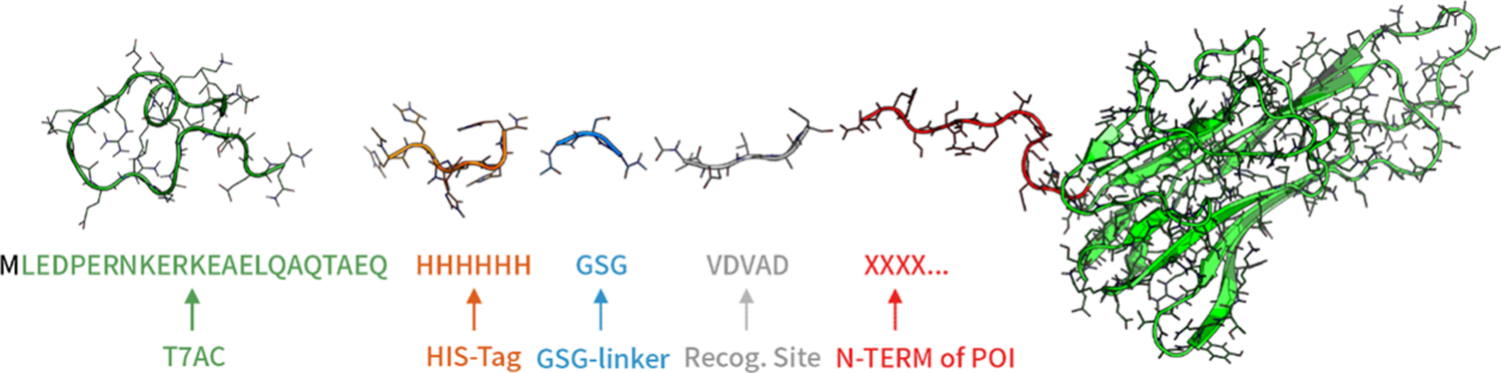
CASPON-tag, attached to the N-terminus of substrate proteins, consists of several components: the T7AC-tag, which serves as a solubility tag and also enhances protein expression, a HIS-tag aiding in protein purification via immobilized metal affinity chromatography (IMAC), and a flexible GSG linker that establishes spatial separation between the HIS-tag and the recognition site. The inclusion of a recognition site enables the binding and cleavage by the CASPON enzyme, allowing for the restoration of the native N-terminus of the protein of interest (POI) after the purification process.

This variant was chosen to be the primary protease in the fusion protein technology, referred to as CASPON (CASPase-based fusiON). The CASPON enzyme is designed to remove the CASPON-tag efficiently and effectively. This tag, illustrated in Figure 2, includes an affinity tag, in this instance, a His-tag consisting of six histidines. On its N-terminal side, the tag is further equipped with a solubility tag, termed the T7AC-tag, which enhances both the solubility and the expression of the tagged protein. A GSG-linker, placed at the C-terminal of the His-tag, creates spatial separation between the His-tag and the recognition site. This recognition site comprises the sequence VDVAD. The CASPON enzyme exhibits high specificity for this site, ensuring efficient binding and cleavage.

To confirm the enhancements of the CASPON enzyme over the cpCasp2, there were further validations through in-silico saturation mutagenesis.^19^ The focus was on the two mutations that were primarily responsible for the improved catalytic efficiency, E105V and G171D. Those sites were mutated through all 20 amino acids to calculate their free energies. The process was reiterated with two different substrates, with Ile and Pro as the P1′ amino acid, respectively, and the in-silico calculations were confirmed experimentally.^20^

The ability of the CASPON enzyme to cleave the CASPON-tag was tested using small peptides and the small protein ubiquitin-conjugating enzyme E2, in which the P1′ amino acid was replaced with all 20 proteogenic amino acids. The cleavability was found to function to a satisfying degree for all amino acids, with some working better than others. However, in larger, more complex proteins, substantial differences were observed, even when the P1′ amino acids were known to work well. For instance, fibroblast growth factor 2 (FGF2) and granulocyte colony-stimulating factor (GCSF) both have an alanine at their P1′ position, yet the cleavage reaction was considerably slower for GCSF.^13^ This discrepancy was hypothesized to arise from a steric effect, prompting the need for further exploration.

To address this, we propose an investigation that utilizes a simulation-based modeling process to evaluate the accessibility of the recognition site for various fusion proteins and the affinity of caspase binding. This approach entails identifying the N-terminus of the POIs and searching for a vast number of possible conformations of these N-termini through molecular dynamics (MD) simulations. A fitting approach is subsequently used to place the conformations into the active site of the caspase. Through this process, we obtain an estimate of the cleavability of a POI based on the number of fitting structures. We aim to gain a more detailed understanding of the factors that govern the interaction of these proteins with the CASPON enzyme, thereby allowing us to further optimize the protein cleavage process. This methodology serves as a proof of principle and can be straightforwardly applied to alternative proteases to remove tags. Consequently, our work contributes to the usability of the CASPON method, providing a quick estimate of the feasibility of the approach. By demonstrating its general applicability, we facilitate the broader adoption of CASPON technology and similar techniques in the field. Furthermore, our modeling approach should offer a simple means to efficiently predict if cleavage by the CASPON technology is likely to be successful *a priori*, allowing for a quick estimate of cleavability within a matter of a few days.

## Methodology

We have studied the cleavability of five distinct proteins of interest (POI): tumor necrosis factor-α (TNFα), granulocyte colony-stimulating factor (GCSF), fibroblast growth factor 2 (FGF2), interferon γ (IFNγ), and a single-chain variable domain of an antibody (scFv). These form a diverse set of proteins with the following N-terminal amino acids: valine (TNFα), alanine (GCSF, FGF2), glutamine (IFNγ), and glutamic acid (scFv), which are all expected to be cleaved efficiently according to the experiments on peptides and the E2 ligase.^13^

**Figure 3:**
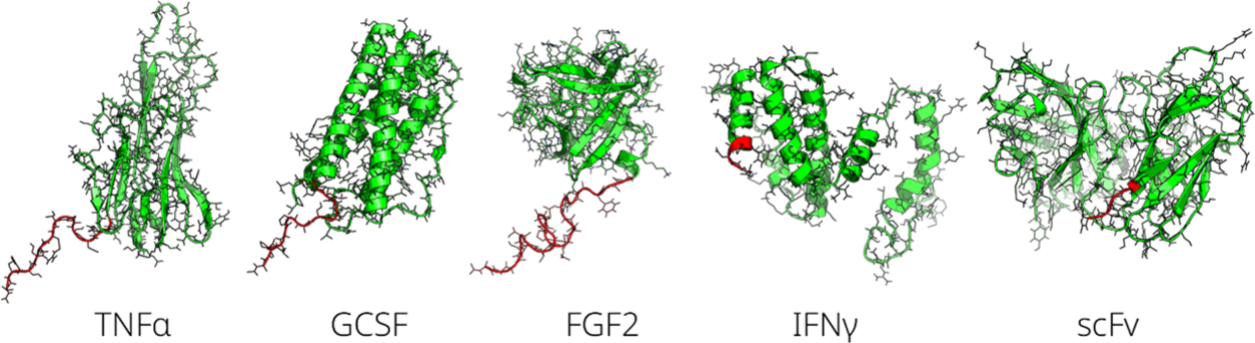
Five proteins of interest (POIs) investigated in this study. Each POI’s N-terminus is highlighted in red, while the bulk of the protein is colored in green. Only the segments identified as the flexible N-terminus were simulated by using molecular dynamics (MD). Following the simulation, a set of 10,000 simulated structures was reattached to the main body of the POIs. Structures that were reintegrated without substantial clashes were clustered into a set of 100 structures. This bundle represents the flexibility of the N-termini of the POIs.

As the N-terminal regions and the CASPON Tag can be considered intrinsically disordered regions of otherwise folded proteins, a fragment-based simulation approach was taken,^21^ followed by conformational selection of cleavage-prone structures. The different steps taken in this approach are summarized in Table 1. In the first step, the flexible portion of the N-terminus for each POI was determined based on a visual inspection using PyMOL and supported by predictions of disorder using SPOT-Disorder 2^22^ (Figure S1, in Supporting Information). In general, all N-terminal amino acids for which no secondary structure was assigned were considered flexible, with a minimum of four amino acids. The minimal size of the N-terminal portion was based on the structure of the caspase-2 active site, which can accommodate nine substrate binding sites in total, five preceding (S5–S1) and four following (S1′–S4′) the cleavage site. Consequently, simulating at least four amino acids, regardless of their occurrence in secondary structure elements, appeared to be an appropriate choice. The determined N-termini of the POIs are illustrated in red in Figure 3. In the simulations, one additional amino acid from the remaining protein was included (see below).

**Table 1:** Outline of Steps Taken for Each POI

1	identify N-terminus of POI
2	perform vacuum MD with SD and LE
3	ligate 10,000 of sampled structures back on bulk of protein
4	apply EM, retaining only low-energy structures
5	cluster the structures, reducing to 100 representative structures.
6	fit N-termini into caspase active site
7	apply EM, retaining low-energy structures
8	evaluate cleavability based on structure fit count

### MD Simulations

Molecular dynamics simulations were performed to generate a large variety of structural models of the N-termini of the POIs and of the different elements of the CASPON tag. All molecular dynamics (MD) simulations were performed using the GROMOS11 simulation software suite.^23^ Starting structures were modeled using MOE 2019.01 (Molecular Operating Environment).^24^ The starting structures modeled for the simulations featured capped ends: a methyl-acetate on the N-terminus and an additional *N*-methyl on the C-terminus. To allow for a time step size of 2 fs, the SHAKE algorithm was used in all simulations to constrain the bond lengths.

#### Simulations of the CASPON-tag Components

Owing to the size of the CASPON-tag and the absence of structural information, its parts (T7AC-tag, His-tag, and GSG-linker) were individually simulated. These simulations were used to visualize the potential conformations of the CASPON-tag on a fusion protein when it is bound to the CASPON enzyme. As such, the CASPON-tag’s recognition site was not part of the simulations, as it is presumed to be in a bound state within the active site. The atomic interactions were described using the GROMOS 54A8 force field parameter set.^25^

##### Simulation Settings

Based on the distribution of negatively charged residues in the sequence of the T7AC tag, it was predicted to be of an α-helical nature. Accordingly, we chose to model its starting conformation as an α-helix. The His-tag and the GSG-linker were modeled in an extended form. Each CASPON-tag component was individually solvated in a water box, where a minimum distance from the solute to the box wall of 1.4 nm was set. Due to the charged nature of the T7AC-tag, three sodium ions were added to counter its negative charges and another ten sodium and ten chloride ions were introduced to result in a salt concentration of 0.08 M. Initial velocities were randomly assigned from a Maxwell–Boltzmann distribution, utilizing distinct initial seeds for each of three independent replicates of each CASPON-tag component. The equilibration followed a 5-step protocol, beginning at an initial temperature of 60 K and incrementing by 60 K at each stage until reaching 300 K, totaling in an equilibration time of 100 ps. The simulations were conducted at constant temperature and pressure using the weak-coupling algorithm with relaxation times of 0.1 ps for temperature and 0.5 ps for the pressure. Positional restraints were applied to all atoms, starting at 2.5 × 10^4^ kJ/mol and reduced by 1 order of magnitude at each protocol step until being fully released in the final step.

The production runs had varied simulation lengths, set to 1000, 300, and 200 ns, for the T7AC-tag, His-tag, and GSG-linker, respectively, reflecting the relative size of the peptides.

#### N-terminus Peptide Simulations

With the aim of rapid and efficient prediction of the cleavability of a novel POI, we used a fast search algorithm based on molecular dynamics simulations for the N-termini. These simulations were performed in vacuum, and atomic interactions were accordingly characterized using the GROMOS 54B7 force-field parameter set,^26^ which is tailored for vacuum simulation applications.

Vacuum simulations, while enabling faster calculations, are not without their challenges, including the risk of getting trapped in local minima due to the absence of solvent-mediated interactions. To ensure fast scanning of the conformational space of the N-termini, we incorporated two strategies: stochastic dynamics (SD) and local elevation (LE).

- SD emulates part of the solvent’s effect by applying random forces and atomic friction to the peptide.^27^ We set the SD temperature to 600 K to accelerate our search for a wide variety of configurations.
- LE biasing, the second technique we employed, was configured to the distance type. We selected the terminal N- and C-atoms within each peptide and a specified number of grid points between them. As the interatomic distance remains at a specific distance in the grid, penalty energies are gradually added at that grid point, thereby encouraging the peptide to sample conformations at a lower-energy grid point.^28^ This promotes continual movement within the peptide, thereby enhancing conformational sampling throughout the simulation.

As a result, these two techniques facilitate the search for possible configurations of the N-termini. While SD is employed from the onset of the equilibration phase, LE is activated after the equilibration phase at the beginning of the production phase.

##### Simulation Settings

The N-terminal peptides were slowly heated up through an equilibration protocol consisting of 10 stages, each consisting of 10,000 steps, resulting in a total simulation time of 200 ps.

The temperature set via SD was increased by 60 K at each stage, initiating at 60 K and finally ending at 600 K. For each of the peptides, an extended conformation was chosen as the starting structure, and for each of 10 independent replicate simulations, a unique initial seed was used for the random assignment of initial velocities. We extended each peptide sequence by incorporating one subsequent amino acid from the full protein sequence to potentially enhance the capture of local effects at the site of ligation of the N-terminus and the rest of the protein.

Positional restraints were used to prevent drastic rearrangements and potential premature folding of the peptides due to their self-interaction in a vacuum. These restraints were gradually relaxed in each stage of the equilibration protocol, starting from 2.5 × 10^4^ kJ/mol, sequentially reducing by a factor 10 for the following 4 stages, and finally releasing them in the final stage.

After the equilibration phase, the LE settings were activated for the production phase with configurations varying across each N-terminus simulation. The setup of LE will be explained based on the settings of the N-terminus of TNFα. The LE parameters for the N-termini of the other POIs can be found in Table 2. The sequence for the N-terminus simulation of TNFα comprised 13 amino acids with the sequence VRSSSRTPSDKPV, 12 of which were identified as flexible. The number of grid points used along the distance was set to 100. The effective range of this grid spanned 0.4 to 4.0 nm. A WLES value of 2.0 designates the width of the local function at each grid point, while an RLES value of 2.5 is the cutoff applied to the range of action of the local function, both measured in units of grid spacing. Each time the peptide occupies a particular distance grid point, a penalty energy of 2.25 × 10^–5^ kJ/mol is added (CLES). The total simulation length for the production phase was 500 ns with a total of 10 replicates. Note that this approach is intended to lead to a fast search of possible conformations without maintaining accurate probability distributions.

**Table 2:** Local Elevation Parameters for All 5 POIs^a^

parameters	TNFα	GCSF	FGF2	IFNγ	scFv
*GRIDMIN* [nm]	0.4	0.4	0.4	0.4	0.4
*GRIDMAX* [nm]	4.0	4.0	7.0	1.7	1.7
*NGRID*	100	100	170	40	40
*CLES* [kJ/mol]	2.25 × 10^–5^	2.25 × 10^–5^	2.8125 × 10^–5^	8.75 × 10^–6^	8.75 × 10^–6^

a Only the parameters that differ across simulations are listed. GRIDMIN and GRIDMAX denote the start and end distances of the LE grid. NGRID sets the number of grid points. CLES is the penalty energy increment added when the interatomic distance remains constant at a specific grid point, thus stimulating ongoing peptide movement.

### Cleavage Predictions

#### Stitching the N-terminus Peptides Back onto the Proteins

**Figure 4:**
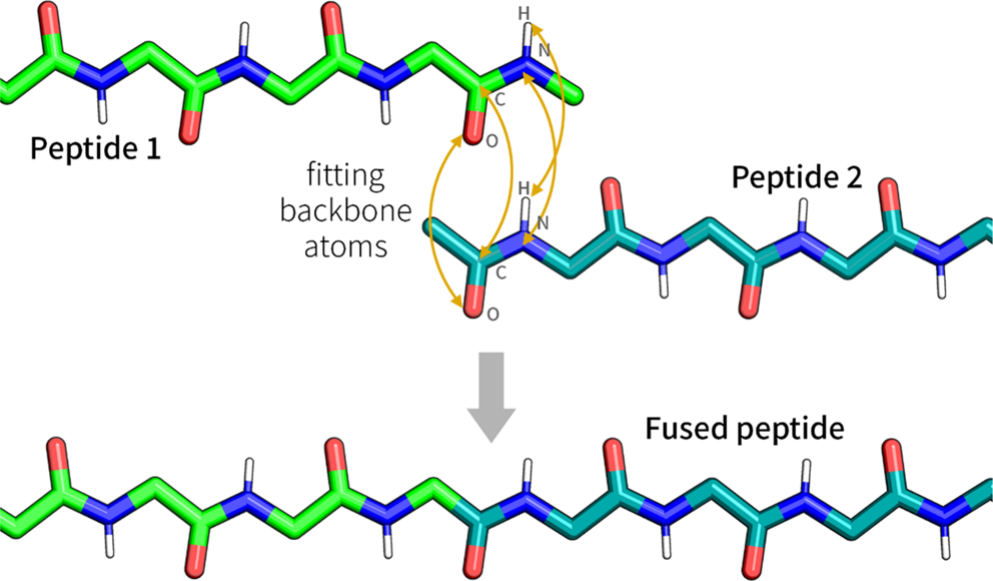
A schematic representation of the fitting and ligation process of the GROMOS++ program, fit_ener_traj. This example shows two peptides with capped ends, allowing fitting on the ends. The peptide 1 atoms of the last residue (C, O, N, H) are fitted with the peptide 2 atoms of the first residue C, O, N, H. Subsequent to the fitting, the atoms of peptide 1 used for the fitting are then replaced with those of peptide 2, including all atoms that are C-terminal from the fitted atoms. This procedure results in a neatly fused peptide, ligated from peptides 1 and 2.

From the trajectory data of all ten replica simulations, 10,000 structures were randomly selected. Using the GROMOS++^29^ program fit_ener_traj, these N-terminal structures were reattached to the main body of the protein, using only the amino acids identified as the flexible part of the N-terminus (with a minimum of four residues). The reattaching worked by fitting 4 backbone atoms of the N-terminus peptide and of the main body to each other, schematically shown in Figure 4. After fitting, we replaced the atoms of the N-terminus peptide after the Cα (i.e., the Cα before the fitted atoms) and inserted the atoms of the main body after the same Cα, yielding 10,000 structures of the main body that is rigid, presenting a wide variety of N-terminal conformations. To accommodate minor atomic clashes, a steepest descent energy minimization with a maximum 100 steps was employed. During this minimization, only the N-terminal atoms were free to move, and the procedure was terminated if the energy changed by less than 0.5 kJ/mol. Any remaining interatomic clashes that could not be resolved by this small energy minimization are identified by large nonbonded interaction energies. Upon recalculation of the nonbonded interaction energies, we retained only structures with negative energies, ranging from 393 to 8570 structures. For these calculations, the GROMOS force field 54B7 was used.

Two approaches were employed to fit the remaining structures into the active site of the caspase-2: the first involved directly using the complete set, i.e., all structures that remained after the N-terminus stitching, while the second involved using a clustered set, defining clusters and utilizing only the central member structure from each cluster for fitting. The specific number of structures for each approach is listed in Table 3. For the clustering method, structures were aligned on the backbone of the bulk of the protein, while a root-mean-square deviation (RMSD) matrix was calculated. This was based on the positions of the backbone atoms (N, Cα, C) of the reattached N-terminus (e.g., in the case of TNFα: residues 1–12). A clustering cutoff was selected so that each POI would be represented by 100 clusters, from which the central member structures were used for subsequent fitting. The second approach was especially beneficial in reducing computational time and aimed to assess whether clustering provides a reliable estimate for cleavage prediction.

**Table 3:** Remaining Structures

POI	remaining structures	clustering cutoff [nm]	num of clusters
*TNFα*	3962	0.418	100
*GCSF*	1339	0.57	100
*FGF2*	8570	0.78	100
*IFNγ*	4694	0.175	100
*scFv*	393	0.175	17

#### Fitting the POIs into the Caspase-2 Active Site

For this stage, we used the previously described structure of caspase-2.^30^ The structure already contains a 9-residue peptide in its active site, which spans from substrate-binding sites S5 to S1 and extends to S1′ to S4′. The cleavage site is situated between the S1 and S1′ sites. The N-termini of the POIs were aligned to the peptide bond atoms between the P1 and P1′ sites, as illustrated in Figure 4. All atoms, starting from the atoms used for fitting and extending to P4′, were replaced with the corresponding atoms from the N-termini of the POIs, including their bulk protein atoms.

An EM was applied, only minimizing the energies of the inserted POI atoms, while excluding the caspase atoms. The algorithm executed a maximum of 100 steps but terminated earlier if the energy change fell below 0.5 kJ/mol.

Lastly, nonbonded interaction energies were evaluated, and only structures with energies below zero were retained for each POI. For these calculations, the GROMOS 54B7 force field was used.

### Experimental Data: Preparation of Experimental Cleavage and Sodium Dodecyl Sulfate–Polyacrylamide Gel Electrophoresis (SDS-PAGE)

For each protein of interest (POI), a caspase cleavage reaction was performed. The caspase used was the CASPON enzyme, as described by Lingg et al.,^13^ and the specific substrates included TNFα, GCSF, FGF2, IFNγ, and scFv, which produced as described by Köppl et al.^14^ The reactions were performed in phosphate-buffered saline (PBS) buffer, pH 7.4, with an enzyme/substrate molar ratio of 1:100. A cystamine solution of 20 mM end concentration was used to stop the reactions after the indicated time.

Because the CASPON-tag was particularly difficult to cleave from scFv, a separate gel with time-course reactions extending up to 48 h were performed to monitor any late-stage cleavage activity.

After the reactions were stopped, the samples were prepared for SDS-PAGE. Each sample was diluted to a concentration of 0.5 g/L using the appropriate dilution factor. For each sample, 15 μL was mixed with 5 μL of 4× NuPage LDS buffer (ThermoFisher Scientific) and 3 μL of 2 M dithiothreitol (DTT). The mixtures were then heated to 95 °C for 10 min to denature the proteins. Subsequently, 15 μL of each denatured sample was loaded into the wells of a 4–12% Bis-TRIS gel, with the exception of the ladder, which was loaded in a volume of 10 μL. The gels were run at 200 V for 45 min.

Upon completion of the gel run, the cleavage yield of the caspase reactions was analyzed via densitometric analysis of fluorescence signals using the Image Lab 6.0.1 software. To quantify the cleavage yield of each protein, fluorescence density contrasted the intensity of the cleaved (product) and uncleaved (substrate) bands within a single odd-numbered lane.

## Results and Discussion

**Figure 5:**
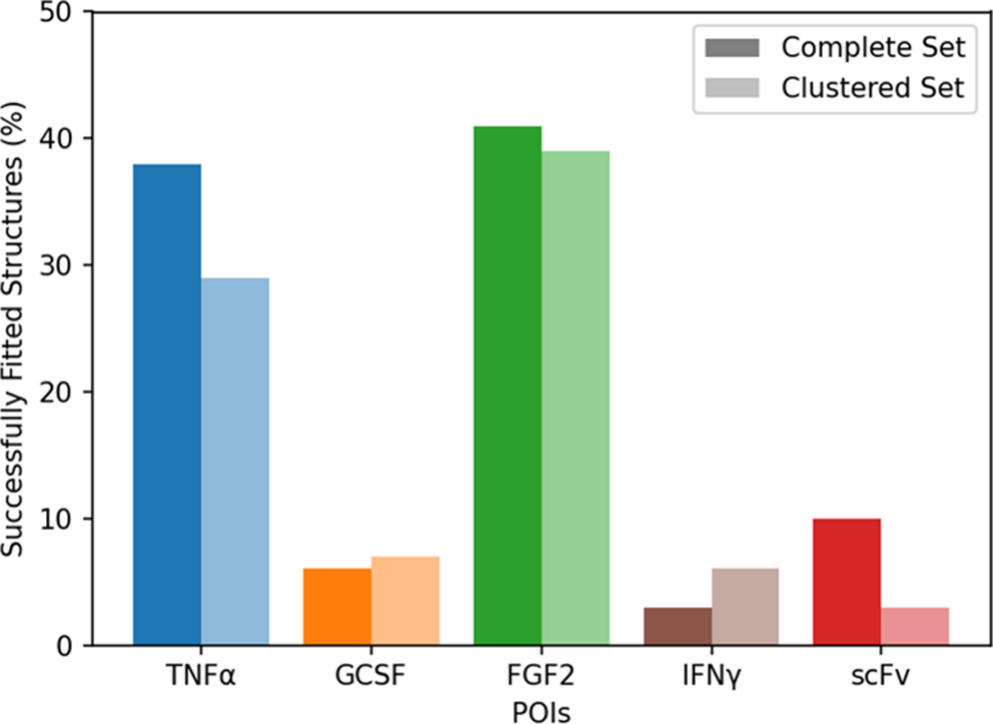
Comparative analysis of successful fittings of POIs was performed using two distinct methodologies. Each POI is represented with a pair of bars: the deeper colors represent the percentage of structures successfully fitted through the complete set approach, while the bar with reduced color intensity indicates the number of clusters successfully fitted using the clustered set approach. Structures were counted as a fit when the interaction energy was below 0. The results give an estimate of how well the cleavage of each POI works.

After the generation of many conformations of the N-termini and reconnecting them to the native protein, 393 to 8570 structures remained, which were clustered as described above (Table 3). Figure S2 in the Supporting Information shows the cumulative distribution of the interaction energy after fitting the POI into the caspase active site. Comparative plots of the fraction of conformations with favorable nonbonded energies after fitting the N-termini into the Caspase 2 for both fitting strategies, with the complete set and the clustered set, are depicted in Figure 5. In the complete set approach, the successful fittings of TNFα and FGF2 conformations show that the majority is compatible with caspase-2 active site, 38 and 41%, respectively. In contrast to that, only 5, 3, and 10% of GCSF, IFNγ, and scFv conformations were successfully fitted. The clustered set revealed similar trends at 29, 6, 39, 6, and 3% for TNFα, GCSF, FGF2, IFNy, and scFv. This seems to confirm that the clustering process did not skew the overall perspective of the fitting. Note that, in contrast to what might be expected from Figure 3, the number of fitting conformations does not simply correlate with the length of the N-terminus. The N-terminus of GCSF is exactly as long as the one of TNFα, yet considerably fewer fitting conformations are found. These patterns offer insight into the steric compatibility of each POI within the caspase active site and, by extension, provide a rough estimate of the efficacy of the cleavage in real-world conditions.

Upon closer examination, while the overall trends between the results of the complete set and those of the clustered set remain consistent, there are subtle differences worth noting. The standout is scFv, which deviates considerably between the two approaches. Given the structural orientation of the scFv’s N-terminus, it is plausible that its unique conformation influences the fitting results. Its N-terminus lies “flat” on the protein surface, allowing for fewer possible ways for its placement, resulting in more structures that are almost identical. This kind of variability underlines the complexity and challenges faced in predicting protein interactions. For scFv, the high similarity among the structures derived from the N-terminus stitching can skew the representation, unavoidably portraying a more favorable fitting scenario than might be the case in reality. This observation may accentuate the advantages of employing clustering prior to active site fitting, possibly giving a more representative sample for the prediction.

**Figure 6:**
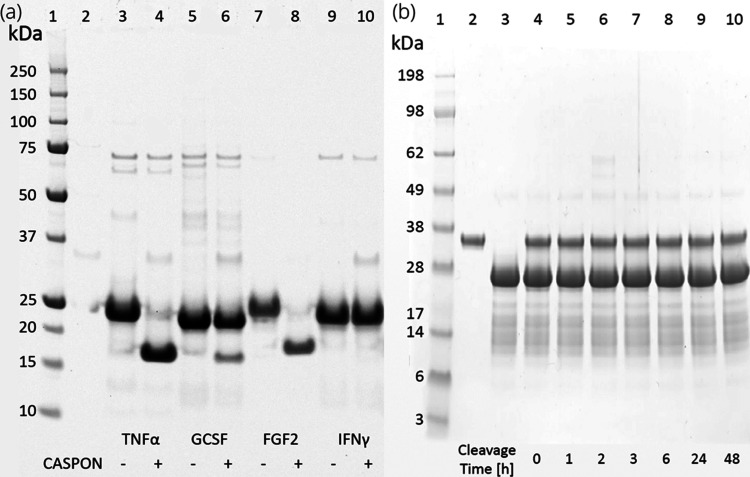
Two SDS-PAGE gels depict the caspase cleavage reactions under various conditions. (a) The first gel shows reactions of four different substrate proteins (TNFα, GCSF, FGF2, and IFNγ). Each substrate is shown in its original state (odd-numbered lanes starting from lane 3) and after 2 h of reaction time with the CASPON enzyme in an enzyme-to-substrate ratio of 1:100 (even-numbered lanes starting from lane 4). Lane 1 shows a kDa scale for orientation, and lane 2 is empty. Lanes are filled with 15 μL of the sample. Cleavage yields were determined by comparing the fluorescence densities of the product and substrate bands between odd- and even-numbered lanes. Fluorescence density was analyzed using Image Lab 6.0.1. (b) Due to particularly challenging cleavage of scFv, a second gel was made, visualizing a time-course reaction of the CASPON enzyme with scFv at a caspase:substrate ratio of 1:10. Lanes 2 and 3 represent the CASPON enzyme and scFv protein separately. The lanes 4 to 10 represent the reaction progress at different time points from 0 to 48 h. All reactions were carried out in PBS, pH 7.4, buffer with a substrate/enzyme molar ratio of 100:1, and stopped after the designated reaction time with a cystamine solution (20 mM end concentration).

When compared to the experimental data, shown in Figure 6 and Table 4, both methods, but in particular the one with the clustered set, appear to offer reliable indicators of POI cleavability (Figure 6). The SDS-PAGE shows nearly full cleavage for TNFα and FGF2, achieving 89 and 94% within 2 h, while GCSF and IFNy exhibit cleavage yields of 21 and 3% in the same time span, respectively.

**Table 4:** POI Cleavage Yields of TNFα, GCSF, FGF2, and IFNγ Calculated from the SDS-PAGE in Figure 6a Based on the Fluorescence Density of the Product and Substrate Bands Between Odd- and Even-Numbered Lanes^a^

POI	cleavage yield [%]
*TNFα*	89
*GCSF*	21
*FGF2*	94
*IFNγ*	3

a All 4 proteins were subjected to 2 h reaction time with the CASPON enzyme in an enzyme-to-substrate ratio of 1:100.

While the percentages of successful fittings do not directly reflect the cleavage yield, they do provide insight into the potential energy barriers associated with the interaction. Using the concept of conformational selection, a higher percentage of successful fittings implies a larger pool of favorable conformations and thus a lower energy penalty for interaction. Conversely, a lower percentage of successful fittings shows that there would be a rather high free energy penalty for the interaction to happen. This is consistent with the observed experimental cleavage yields, as seen in the cases of TNFα and FGF2, as opposed to GCSF, IFNy, and scFv.

We further looked at the interaction energies between the POIs and the caspase of successfully fitted conformations. These are calculated by considering only the interactions of the atoms of caspase-2 with the atoms of each POI, excluding the N-terminus. These numbers should give insights into the compatibility of the POI positions, as determined by our fitting process.

**Figure 7:**
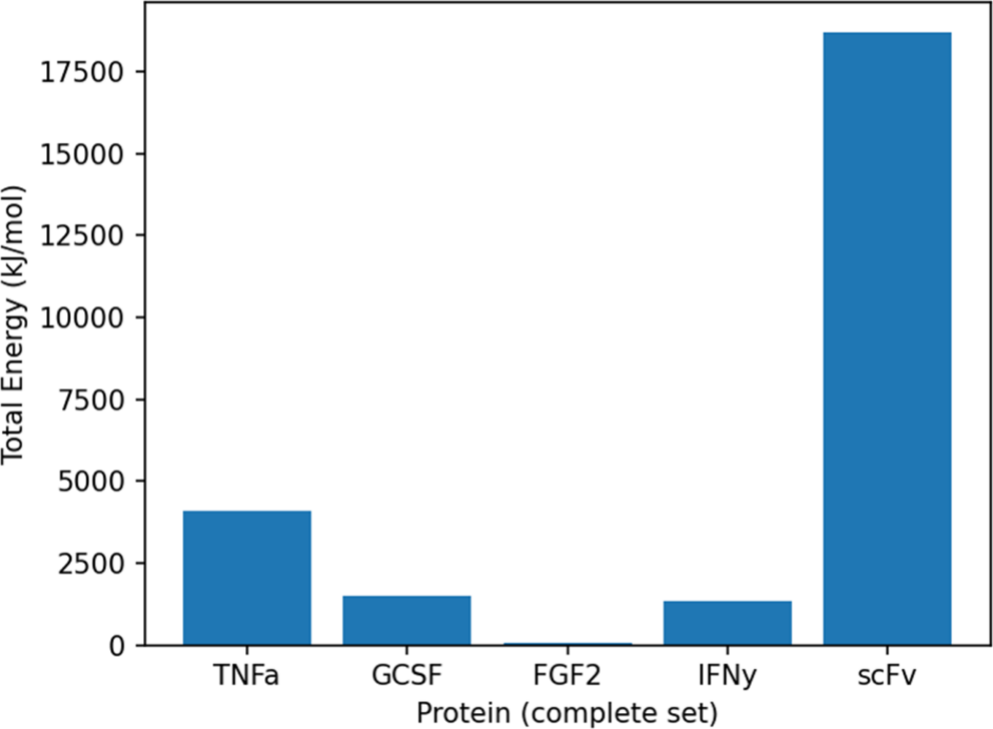
Energies of the complete set: median interaction energies between caspase-2 and POI (excluding N-terminal atoms) for all 5 POIs. Only the energies between caspase-2 and the POI atoms are considered.

We can see in Figure 7 that for the fitting approach using the complete set, scFv stands out, exhibiting high interaction energies, indicating a rather unfavorable fitting. This is consistent with the results obtained from the SDS-PAGE experiment, where scFv proved to be resistant to cleavage. The much lower interaction energies for TNFα, GCSF, FGF2, and IFNγ, in contrast, demonstrate more favorable fittings, albeit to varying degrees.

**Figure 8:**
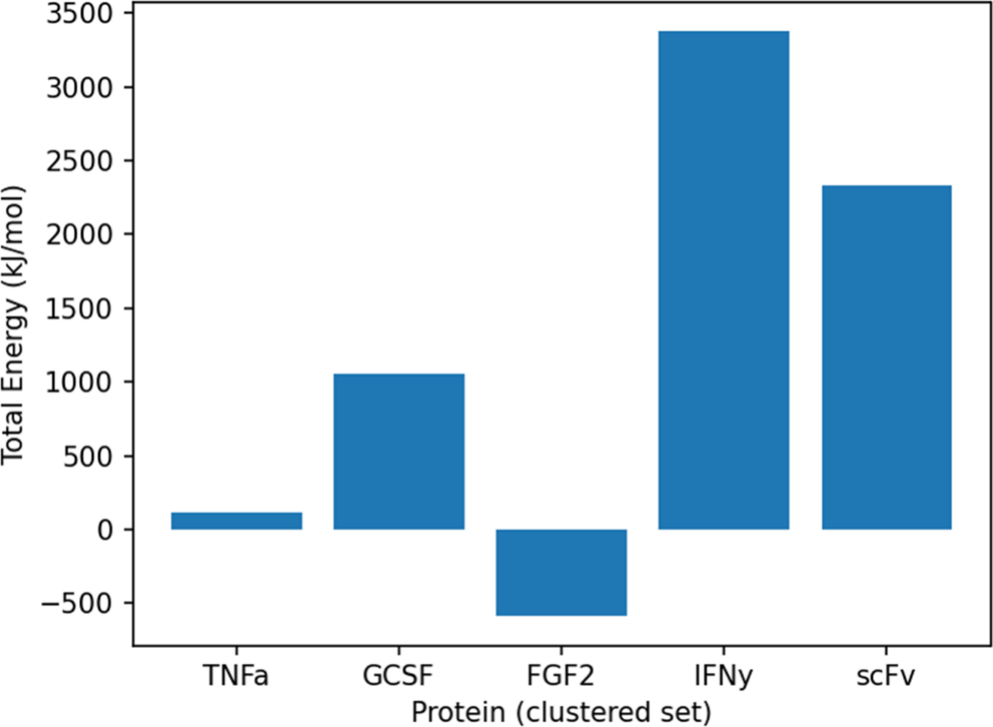
Energies of the clustered set: median interaction energies between caspase-2 and POI (excluding N-terminal atoms) for all 5 POIs. Only the energies between caspase-2 and the POI atoms are counted.

With the fitting approach that utilizes only the clustered set, as depicted in Figure 8, we observe that FGF2 and TNFα maintain low energy values. These low values correlate well with the ease of cleavability in the SDS-PAGE.

The other three proteins, GCSF, scFv, and IFNγ, showed higher median interaction energies, particularly those of the latter two. This disparity suggests that even though within the successfully fitted structures, these three proteins seem to be less sterically compatible in the caspase-2 active site compared to FGF2 and TNFα, reaffirming the relationship between the interaction energies and the cleavability.

**Figure 9:**
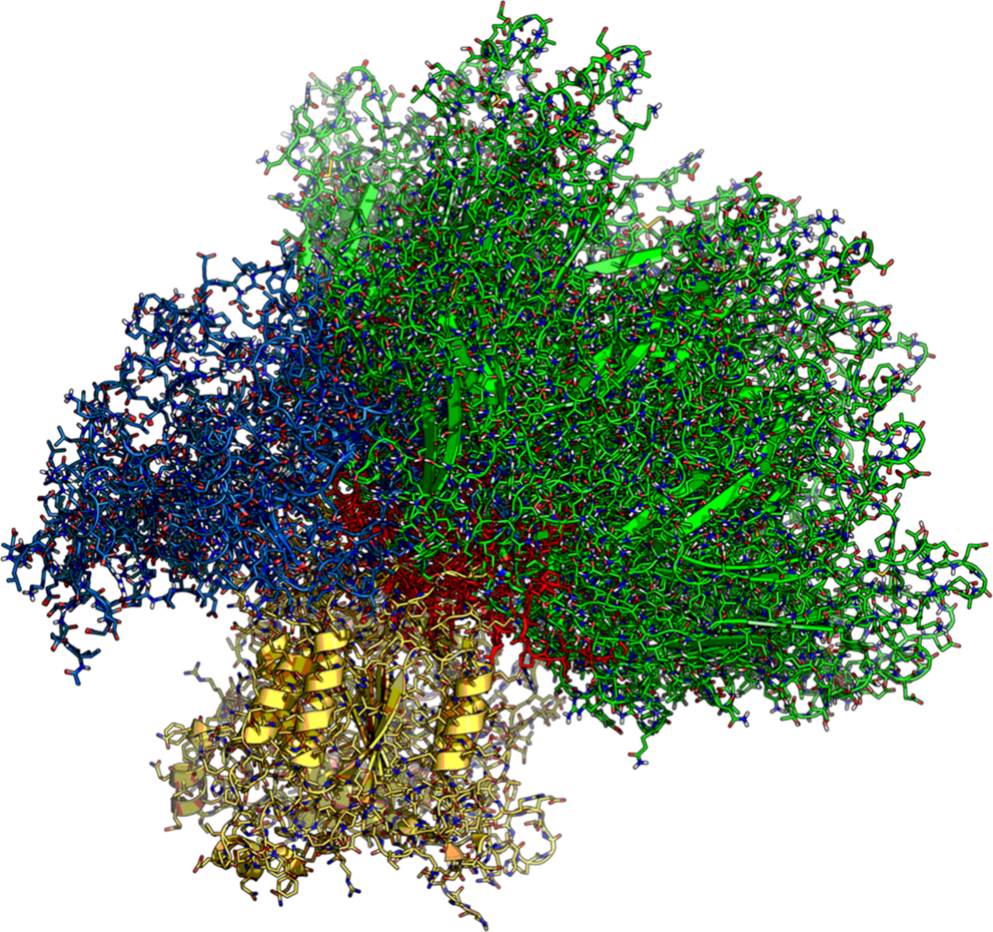
A visual representation of all tagged TNFα structures complexed to the active site of the CASPON enzyme superimposed on the CASPON enzyme structures. This illustrates the dynamic range of movement available to the protein and the CASPON-tag while in a bound state. The CASPON enzyme is colored yellow, while the substrate protein TNFα is colored blue (CASPON-tag), red (flexible N-terminus portion), and green (bulk of the protein).

The dynamic interaction of CASPON-tagged TNFα with the CASPON enzyme active site is shown in a visual representation in Figure 9. It depicts all 38 successfully fitted TNFα structures, with the addition of the CASPON-tag for visual representation, as it is bound to the active site. By superimposing the CASPON enzyme structures, we can distinctly see the relative mobility and positional diversity of the TNFα structures. The visual representations for the other POIs can be found in the Supporting Information (Figure S3).

The simulations of the N-termini used for the cleavage predictions were performed in a vacuum with enhanced sampling techniques for a fast evaluation process, as our method is designed to provide results within a few days. Simulations in explicit water, while more accurate, require vastly more computational time, which is especially true for longer N-termini. In fact, the vacuum simulations can be readily run on older compute nodes without the support of GPUs. To assess the validity of our vacuum simulations, we conducted 5 replicates of 100 ns simulations of the N-termini of each POI in explicit water. We then performed joint clustering of the structures obtained from both water and vacuum simulations.

Our analysis, detailed in Figures S4–S8 of the Supporting Information, demonstrates that the majority of conformations observed in water were also present in the vacuum simulations. The clustering was based on an all-against-all RMSD matrix, and a cutoff was used to ensure that the first 100 clusters covered 80% of all structures. We found that for each of these first 100 clusters, the clusters were made up of both water and vacuum conformations. This leads us to believe that the enhanced sampling vacuum simulations do not omit any significant conformations, validating our approach for these quick predictions. Beyond the first 100 clusters, we observed more clusters populated with only vacuum simulation conformations, suggesting that the water simulations have not sampled the entire conformational space.

## Conclusions

We investigated the cleavability disparities seen in proteins by the CASPON enzyme, specifically when the P1′ amino acid is known to exhibit satisfactory cleavage in smaller peptide systems. Through a simulation-based modeling approach to explore the N-terminal conformational space of each POI and a subsequent fitting method, we evaluated the steric accessibility of the cleavage site.

Our findings were based on two methods for the caspase-2 fittings: an analysis of all conformations obtained from the N-terminus stitching and a second method that utilized clustered conformations. The latter methodology offered several advantages, including normalizing the number of structures for each POI, removing redundant conformations, and increasing the speed of the approach, while not skewing the final results.

Our findings demonstrated a clear correlation between the percentages of successful fittings and experimental cleavage yields when taking conformational selection into account. Specifically, TNFα and FGF2 exhibited relatively high percentages of successful fittings, which corresponded well with their high cleavage yields observed experimentally. In contrast, GCSF, IFNy, and scFv showed lower percentages of successful fittings, aligning with their lower cleavage yields. This correlation showed that our modeling approach is valid for quickly predicting the cleavability of larger proteins by the CASPON enzyme.

In conclusion, we have not only gained valuable insights into the steric accessibility and the interaction energies associated with protein cleavage by the CASPON enzyme but also established a comprehensive workflow that can be readily applied to evaluate new POIs, streamlining the process of understanding their cleavability and interactions with the CASPON enzyme.

## Data Availability

The data underlying this study are openly available in Zenodo at https://doi.org/10.5281/zenodo.10696797, which includes all input files, results, and structure bundles generated from attaching the flexible N-terminus parts onto proteins.

## Supporting Information Available

SPOT disorder predictions for the proteins of interest; cumulative distribution of the interaction energy after fitting the POI into the caspase active site; visual representations for all proteins of interest; combined clustering of simulations in vacuum and in explicit solvent for all five N-termini of the proteins of interest (PDF)
