# Supplementary material for: Caspase-Based Fusion Protein Technology: Substrate Cleavability Described by Computational Modeling and Simulation
Source: J Chem Inf Model. 2024 Jul 1;64(14):5691–700. doi: 10.1021/acs.jcim.4c00316 (PMC11267566; doi:10.1021/acs.jcim.4c00316)
Supplement: Supplementary file 1 — ci4c00316_si_001.pdf [file ci4c00316_si_001.pdf]

Supporting Information for

Caspase-Based Fusion Protein Technology: Substrate Cleavability Described by Computational Modeling and Simulation

Jakob Liu,[1,2] Andreas Fischer,[1,3] Monika Cserjan-Puschmann, [1,3] Nico Lingg,[1,3] Chris Oostenbrink[2,4*]

1) Austrian Centre of Industrial Biotechnology, Muthgasse 18, Vienna, Austria
2) Institute of Molecular Modeling and Simulation, University of Natural Resources and Life Sciences, Vienna (BOKU), Muthgasse 18, Vienna, Austria.
3) Department of Biotechnology, Institute of Bioprocess Science and Engineering, University of Natural Resources and Life Sciences, Vienna (BOKU), Muthgasse 18, Vienna, Austria
4) Christian Doppler Laboratory for Molecular Informatics in the Biosciences, University of Natural Resources and Life Sciences, Vienna. Muthgasse 18, 1190 Vienna, Austria

* Corresponding author. E-mail: chris.oostenbrink@boku.ac.at

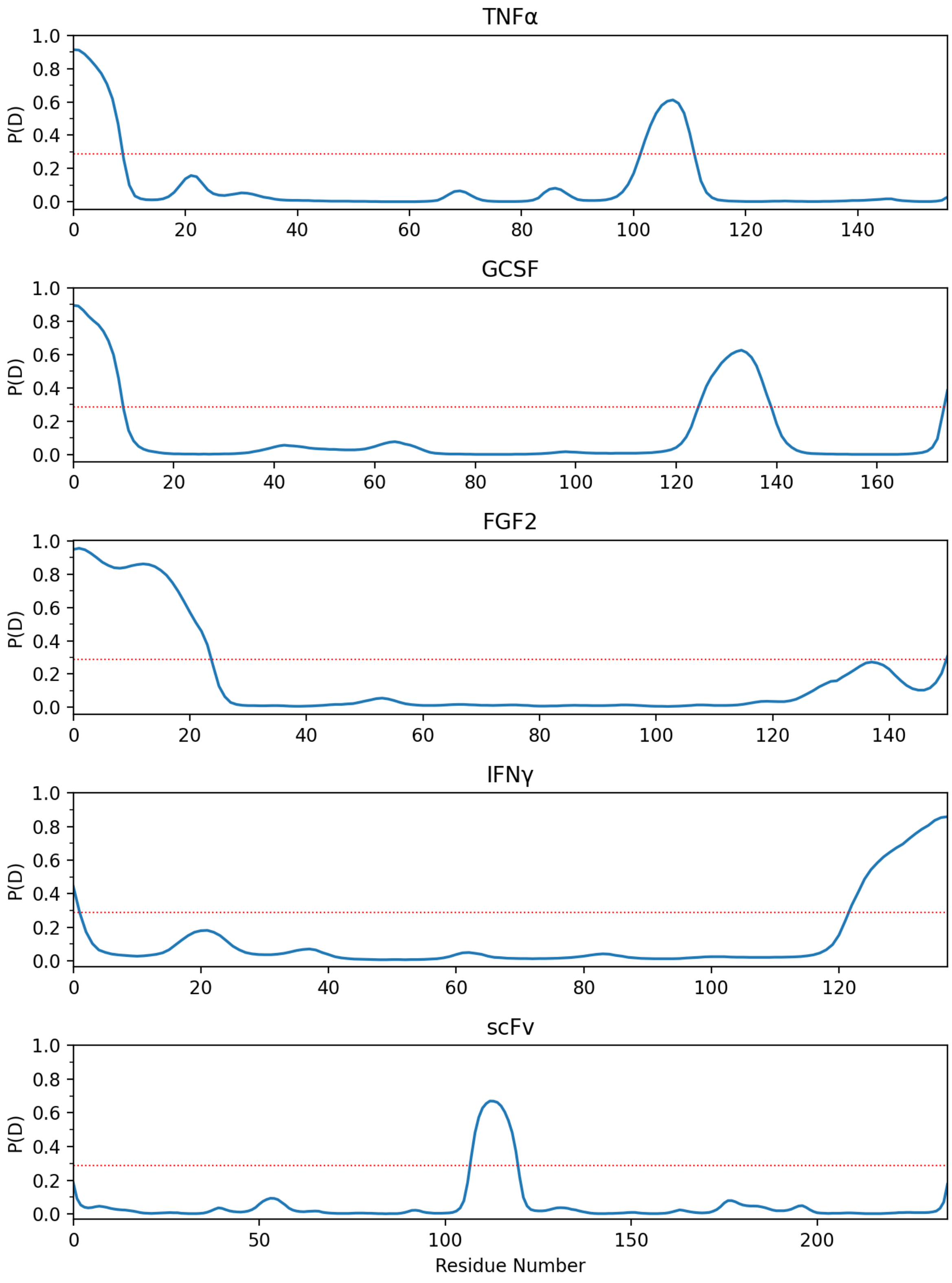

Figure S1. The probability of residues in five different proteins—TNFα, GCSF, FGF2, IFNγ, and scFv—being disordered is illustrated, analyzed by the SPOT-Disorder 2 software. Each subplot shows a protein's amino acid sequence, with the horizontal axis showing residue numbers and the vertical axis depicting the probability of disorder (P(D)). The red horizontal line at P(D)=0.287 indicates a suggested threshold for differentiating between ordered and disordered regions within the protein sequences, as found in the literature.

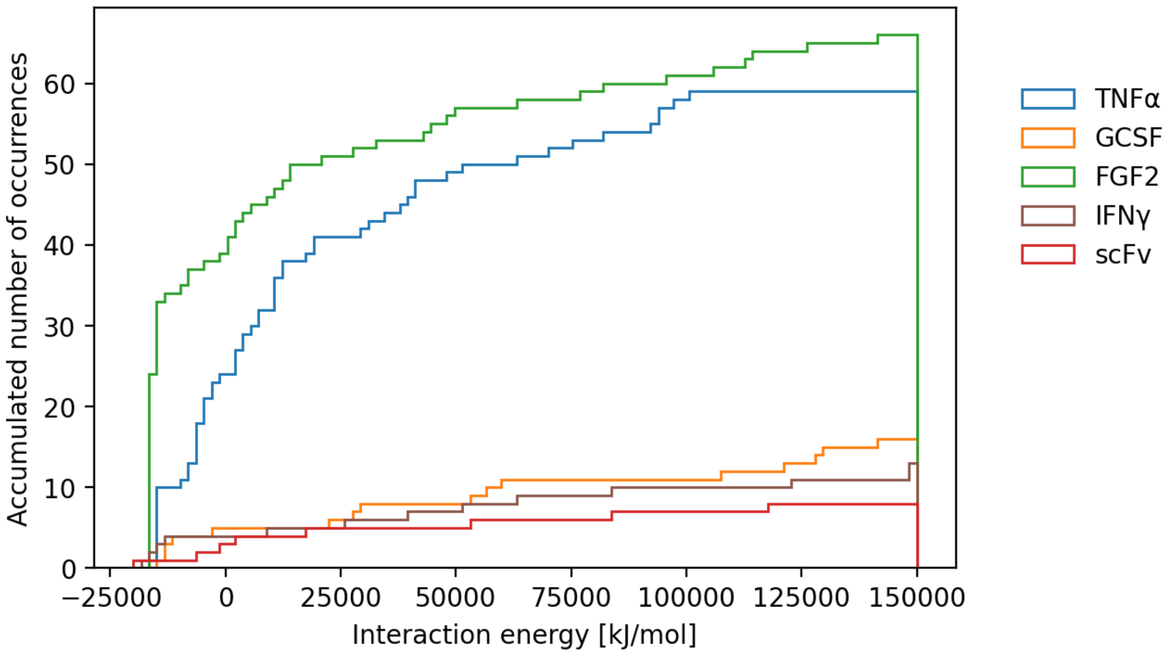

Figure S2. Cumulative frequency of occurrence for each protein — TNFα, GCSF, FGF2, IFNγ, and scFv — against their respective interaction energies with the caspase-2 active site. These interaction energies provide insight into the steric compatibility of each POI within the caspase active site, informing on the potential efficiency of cleavage under real-world conditions. Notably, the variance in fitting success among the proteins does not necessarily correlate with the length of the 'flexible' N-terminus.

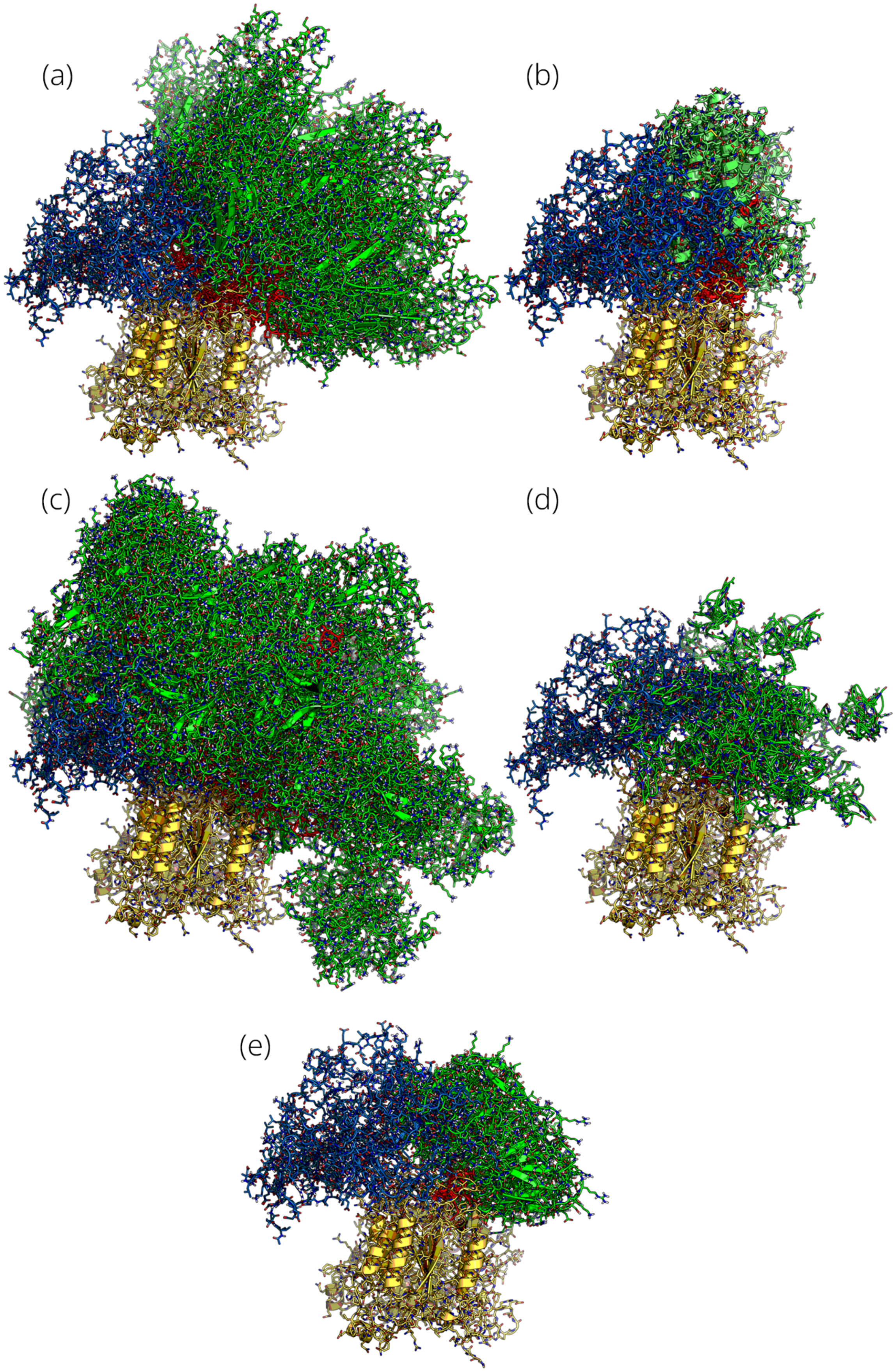
(a)
(b)
(c)
(d)
(e)

Figure S3. A visual representation of all tagged POI structures (a: TNFα, b: GCSF, c: FGF2, d: IFNγ, e: scFv) complexed to the active site of the CASPON enzyme, superimposed on the CASPON enzyme structures. This illustrates the dynamic range of movement available to the protein and the CASPON-tag while in a bound state. The CASPON enzyme is coloured in yellow, while the POIs are coloured in blue (CASPON-tag), red (flexible N-terminus portion) and green (bulk of the protein)

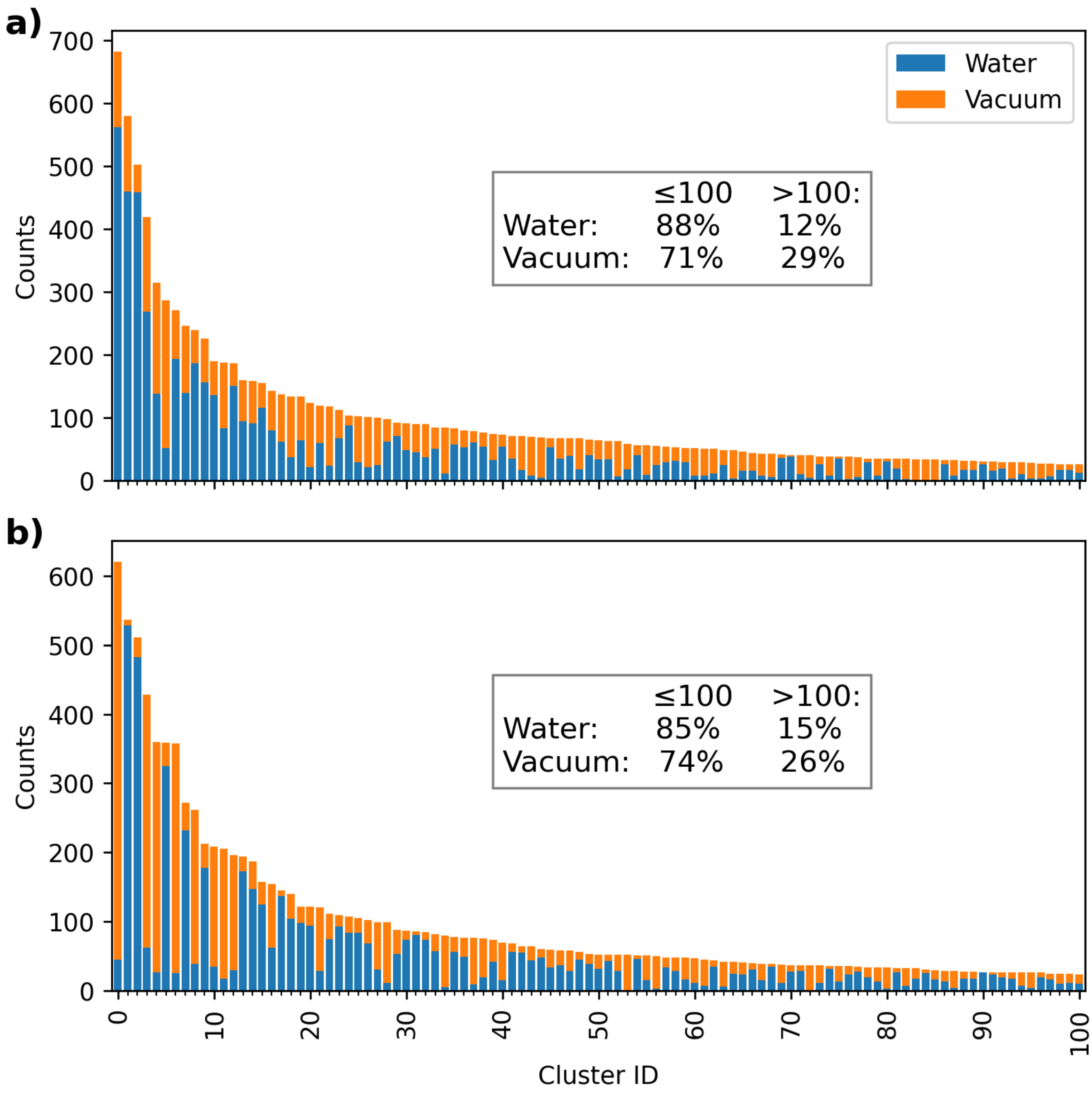

Figure S4. Stacked bar plots illustrating the distribution of structures from joint clustering of water and vacuum simulations for TNF-α. Two types of clustering are compared: (a) 6250 structures from 5x 100 ns water simulations and 10x 100 ns vacuum simulations, and (b) 6250 structures from 5x 100 ns water simulations and 10x 500 ns vacuum simulations. Clustering was performed with a cutoff chosen to ensure the first 100 clusters capture 80% of all conformations, emphasizing the most prevalent conformations. The plots demonstrate that all conformations observed in the 100 ns water simulations are also present in the vacuum simulations, confirming that vacuum simulations do not omit any significant structures. Additionally, the vacuum simulations exhibit a higher diversity of rare structures, as indicated by the percentages of structures within the first 100 clusters and those beyond 100 (inset).

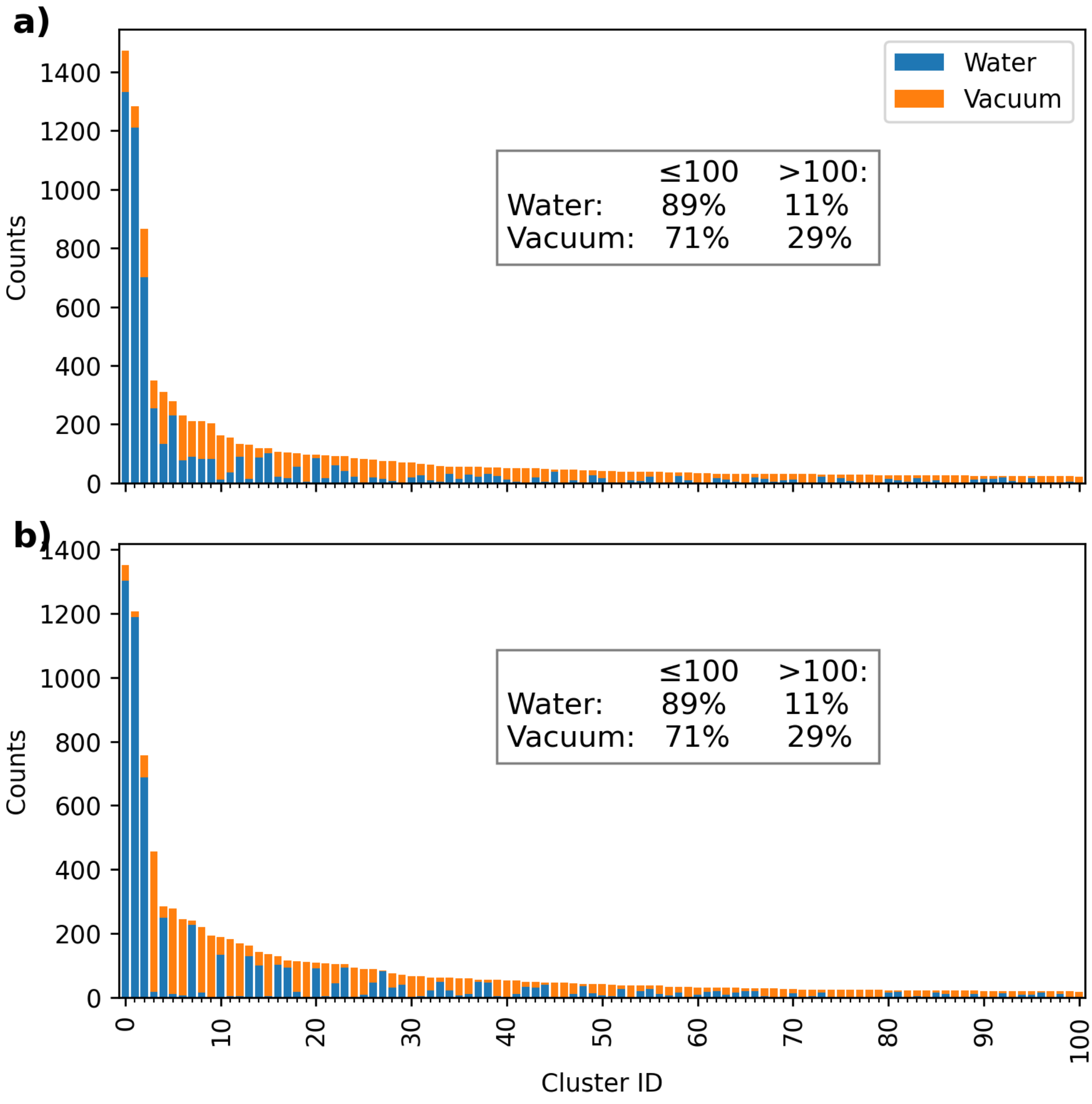

Figure S5 Stacked bar plots illustrating the distribution of structures from joint clustering of water and vacuum simulations for GCSF. Two types of clustering are compared: (a) 6250 structures from 5x 100 ns water simulations and 10x 100 ns vacuum simulations, and (b) 6250 structures from 5x 100 ns water simulations and 10x 500 ns vacuum simulations. Clustering was performed with a cutoff chosen to ensure the first 100 clusters capture 80% of all conformations, emphasizing the most prevalent conformations. The plots demonstrate that all conformations observed in the 100 ns water simulations are also present in the vacuum simulations, confirming that vacuum simulations do not omit any significant structures. Additionally, the vacuum simulations exhibit a higher diversity of rare structures, as indicated by the percentages of structures within the first 100 clusters and those beyond 100 (inset).

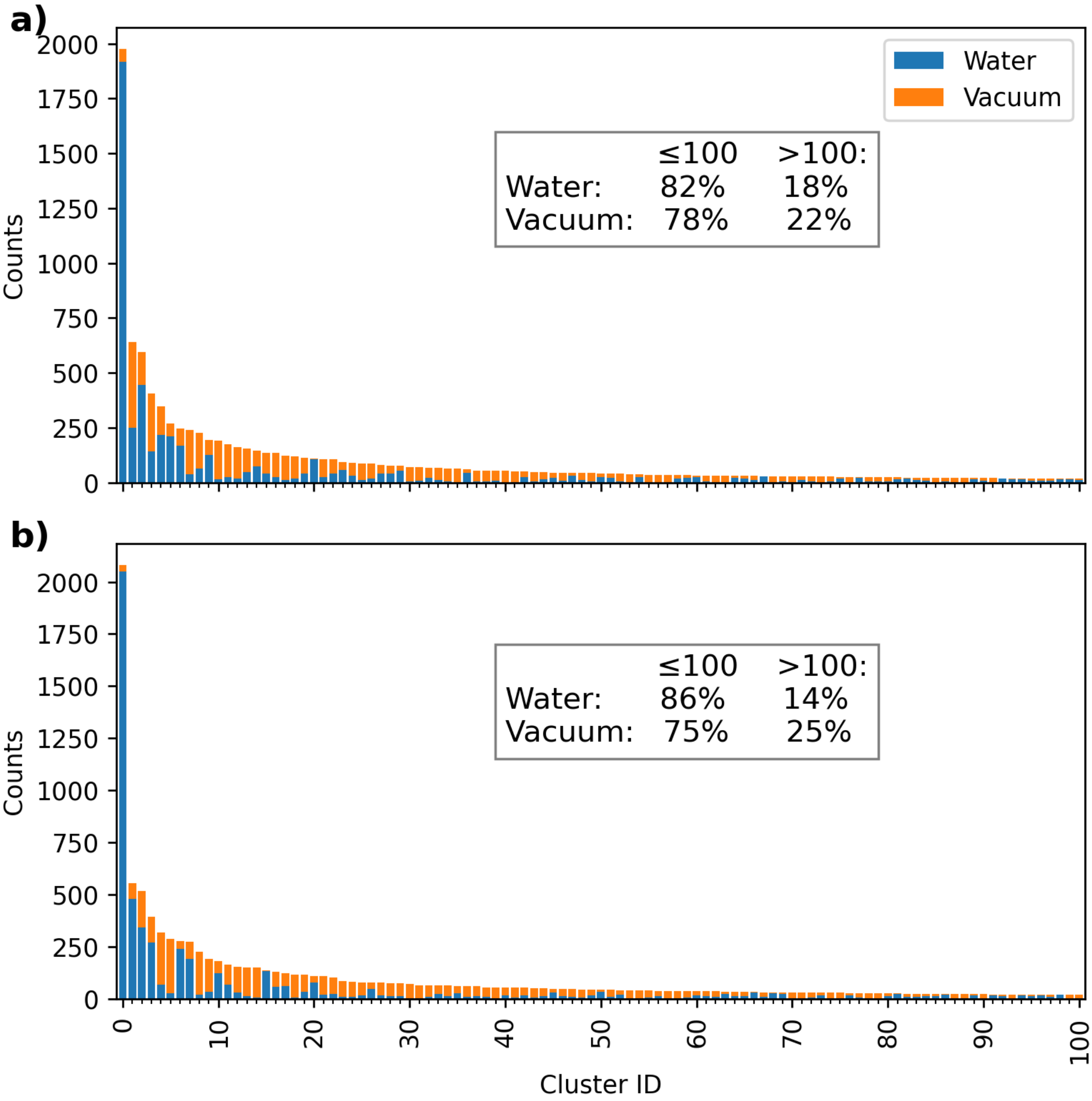

Figure S6 Stacked bar plots illustrating the distribution of structures from joint clustering of water and vacuum simulations for FGF2. Two types of clustering are compared: (a) 6250 structures from 5x 100 ns water simulations and 10x 100 ns vacuum simulations, and (b) 6250 structures from 5x 100 ns water simulations and 10x 500 ns vacuum simulations. Clustering was performed with a cutoff chosen to ensure the first 100 clusters capture 80% of all conformations, emphasizing the most prevalent conformations. The plots demonstrate that all conformations observed in the 100 ns water simulations are also present in the vacuum simulations, confirming that vacuum simulations do not omit any significant structures. Additionally, the vacuum simulations exhibit a higher diversity of rare structures, as indicated by the percentages of structures within the first 100 clusters and those beyond 100 (inset).

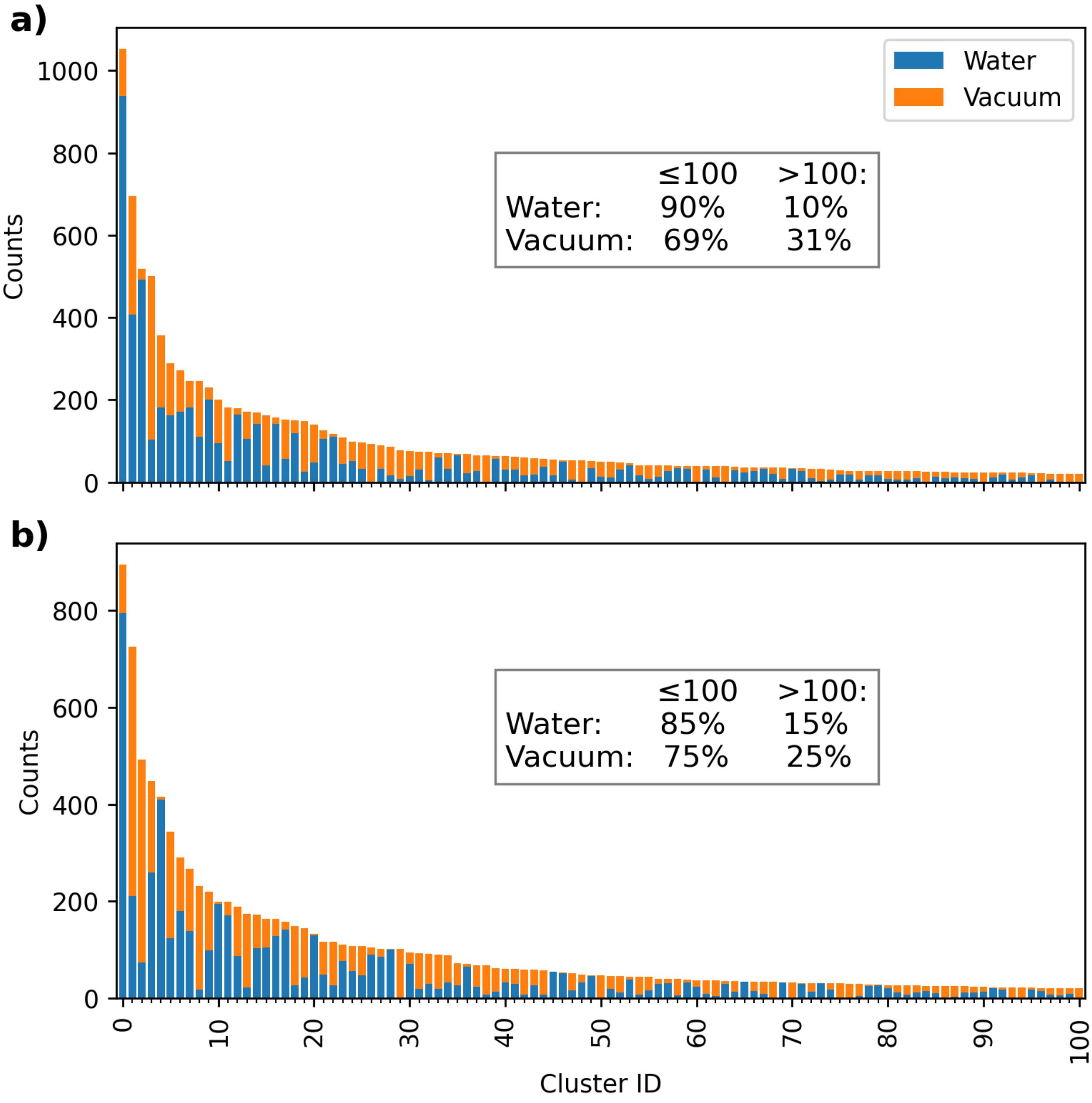

Figure S7 Stacked bar plots illustrating the distribution of structures from joint clustering of water and vacuum simulations for IFNγ. Two types of clustering are compared: (a) 6250 structures from 5x 100 ns water simulations and 10x 100 ns vacuum simulations, and (b) 6250 structures from 5x 100 ns water simulations and 10x 500 ns vacuum simulations. Clustering was performed with a cutoff chosen to ensure the first 100 clusters capture 80% of all conformations, emphasizing the most prevalent conformations. The plots demonstrate that all conformations observed in the 100 ns water simulations are also present in the vacuum simulations, confirming that vacuum simulations do not omit any significant structures. Additionally, the vacuum simulations exhibit a higher diversity of rare structures, as indicated by the percentages of structures within the first 100 clusters and those beyond 100 (inset).

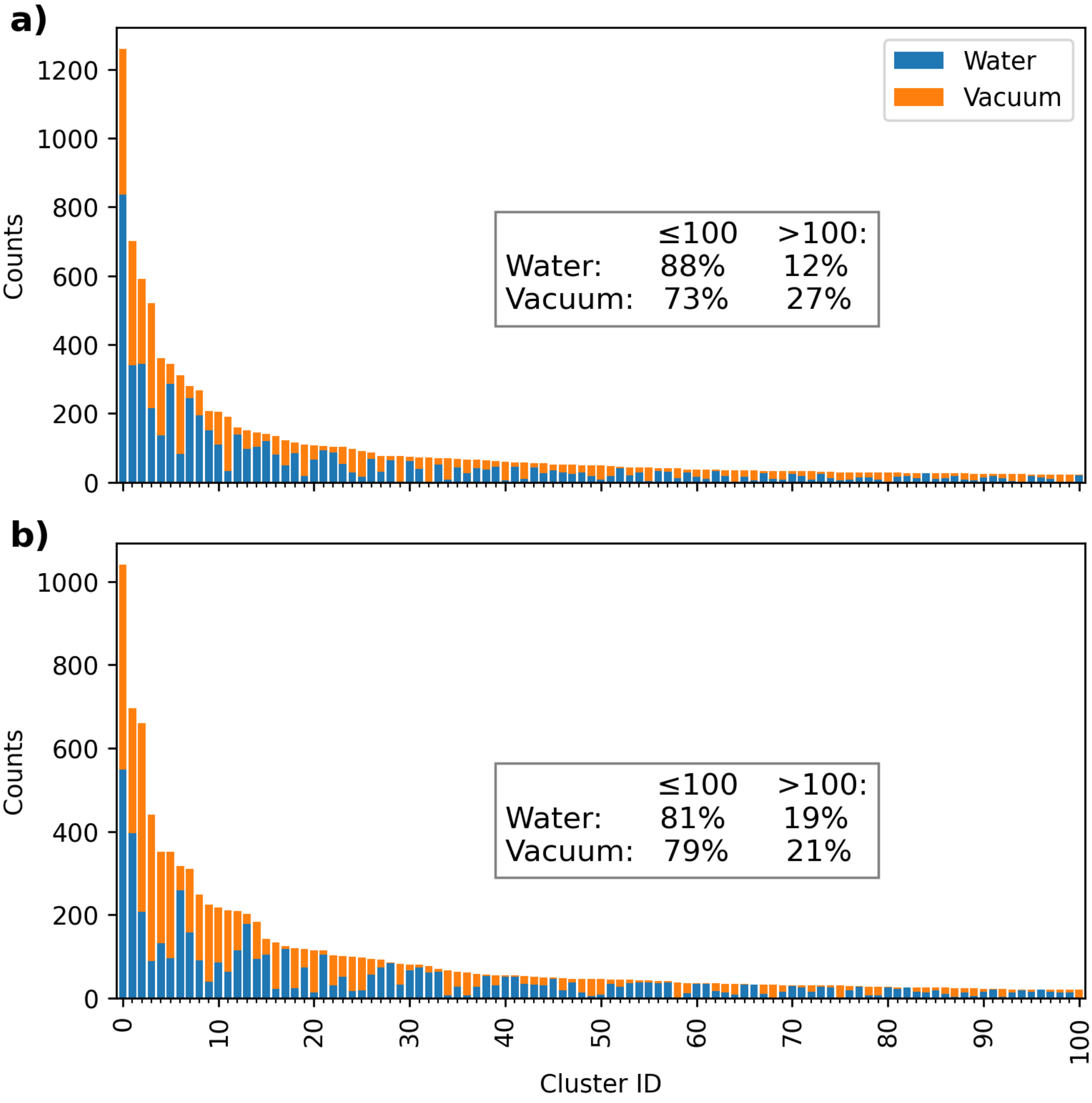

Figure S8 Stacked bar plots illustrating the distribution of structures from joint clustering of water and vacuum simulations for scFv. Two types of clustering are compared: (a) 6250 structures from 5x 100 ns water simulations and 10x 100 ns vacuum simulations, and (b) 6250 structures from 5x 100 ns water simulations and 10x 500 ns vacuum simulations. Clustering was performed with a cutoff chosen to ensure the first 100 clusters capture 80% of all conformations, emphasizing the most prevalent conformations. The plots demonstrate that all conformations observed in the 100 ns water simulations are also present in the vacuum simulations, confirming that vacuum simulations do not omit any significant structures. Additionally, the vacuum simulations exhibit a higher diversity of rare structures, as indicated by the percentages of structures within the first 100 clusters and those beyond 100 (inset).
